# Comparable real‐world effectiveness between switches to cabotegravir + rilpivirine long‐acting or modern daily oral regimens in the United States: an OPERA cohort study

**DOI:** 10.1002/jia2.70068

**Published:** 2025-12-17

**Authors:** Ricky K. Hsu, Michael G. Sension, Jennifer S. Fusco, Laurence Brunet, Quateka Cochran, Brooke Levis, Gayathri Sridhar, Vani Vannappagari, Jean Van Wyk, Michael B. Wohlfeiler, Gregory P. Fusco

**Affiliations:** ^1^ AIDS Healthcare Foundation New York New York USA; ^2^ NYU Langone Health New York New York USA; ^3^ CAN Community Health Fort Lauderdale Florida USA; ^4^ Epividian Raleigh North Carolina USA; ^5^ AIDS Healthcare Foundation Fort Lauderdale Florida USA; ^6^ ViiV Healthcare Durham North Carolina USA; ^7^ ViiV Healthcare London UK; ^8^ AIDS Healthcare Foundation Miami Florida USA

**Keywords:** real‐world evidence, HIV, long‐acting injectable, cabotegravir, rilpivirine, persistence, virologic suppression

## Abstract

**Introduction:**

Cabotegravir + rilpivirine long‐acting (CAB+RPV LA) injectable was approved in the United States in 2021 for HIV‐1 treatment in virologically suppressed (viral load [VL] <50 copies/mI individuals. In clinical trials, CAB+RPV LA was non‐inferior to oral antiretroviral therapy (ART) regimens in virologically suppressed individuals. We compared real‐world effectiveness between CAB+RPV LA and oral ART regimens and assessed predictors of confirmed virologic failure (CVF) on CAB+RPV LA.

**Methods:**

From the OPERA^®^ cohort, ART‐experienced, virologically suppressed adults with HIV switching to CAB+RPV LA or a new oral ART regimen between 21 January 2021 and 31 December 2022 were followed through 30 June 2023. CVF was defined as 2 VL ≥200 copies/ml or 1 VL ≥200 copies/ml + discontinuation. Logistic regression was used to assess CVF risk by regimen and CVF predictors among CAB+RPV LA users.

**Results:**

During the study period, 1362 virologically suppressed adults switched to CAB+RPV LA, and 2783 switched to a new oral ART regimen (92% second‐generation integrase inhibitor [INSTI]‐based). Compared to oral ART users, CAB+RPV LA users were younger, on their prior regimen less time and more likely to switch from an INSTI; median CD4 counts at initiation were similar. At study end, 81% of CAB+RPV LA users and 80% of oral ART users were on their respective regimens. CVF risk with CAB+RPV LA did not statistically differ compared to oral ART (adjusted odds ratio: 0.64; 95% confidence interval [CI]: 0.34, 1.14). Among CAB+RPV LA users, only baseline CD4 predicted CVF; every 100 CD4 cells/µl increase was associated with 20% lower CVF risk (OR [95% CI]: 0.80 [0.64, 0.97]).

**Conclusions:**

In the United States, routine clinical care, CVF risk did not differ between a switch to CAB+RPV LA or new oral ART, with most individuals remaining on their regimens at study end. Lower CD4 count at initiation was the only predictor of CVF on CAB+RPV LA.

## 1 INTRODUCTION

Advancements in antiretroviral therapy (ART) over the past three decades have produced a marked reduction in both morbidity and mortality associated with HIV acquisition [1]. To further improve HIV treatment options, recent clinical developments have focused on long‐acting formulations.

CAB+RPV LA is a two‐drug co‐packaged product of cabotegravir, an HIV‐1 integrase strand transfer inhibitor (INSTI), and rilpivirine, an HIV‐1 non‐nucleoside reverse transcriptase inhibitor (NNRTI) in the United States. CAB+RPV LA is the first and currently the only complete long‐acting injectable ART regimen approved for the treatment of HIV‐1 in the United States [2]. It has been approved for individuals with a viral load (VL) < 50 copies/ml, on a stable ART regimen, and who have no history of treatment failure and no known or suspected resistance to either cabotegravir or rilpivirine [2]. A monthly dosing schedule was approved by the FDA in January 2021 [2], and an every 2‐month dosing schedule was approved in February 2022. As of March 2022, CAB+RPV LA can be initiated with or without an oral lead‐in [3]. The CAB+RPV LA injection window is up to 7 days before or after the target date of the scheduled injection [4].

Clinical trials have demonstrated that CAB+RPV LA injectable (both monthly and every 2‐months dosing) is non‐inferior to daily oral three‐drug regimens [5−12], and that the every 2‐months dosing is non‐inferior to monthly dosing [13−15]. Recently, the data safety monitoring board of the LATITUDE trial stopped the study early after interim analyses demonstrated superiority in virologic effectiveness for CAB+RPV LA versus daily oral ART in individuals with a history of ART adherence challenges [16]. Recent observational studies, including data from 2021 to 2023, have found that most individuals on CAB+RPV LA regimen achieve or maintain virologic suppression and that confirmed virologic failure (CVF) is rare [17−19].

While clinical and observational studies have established the efficacy, safety and tolerability of the CAB+RPV LA regimen, little is known about how outcomes compare in individuals receiving the CAB+RPV LA regimen versus a new oral ART regimen in real‐world clinical settings. Therefore, the objectives of this study were to compare real‐world effectiveness after a switch to CAB+RPV LA versus a new oral ART regimen and to assess the predictors of CVF on CAB+RPV LA over the first 2 years of CAB+RPV LA availability.

## 2 METHODS

### 2.1 Study population

This was an observational cohort study using prospectively captured, routine clinical data from electronic health records (EHRs) from the Observational Pharmaco‐Epidemiology Research & Analysis (OPERA^®^) cohort, which consists of data from 101 clinics in 23 states and territories and includes approximately 14% of people with HIV in the United States. OPERA complies with all HIPAA and HITECH requirements, which expand upon the ethical principles detailed in the 1964 Declaration of Helsinki. OPERA has received annual institutional review board approval by Advarra IRB, including a waiver of informed consent and authorization for use of protected health information (Pro00023648).

The study population included people with HIV aged 18 years or older who were ART‐experienced, had a VL < 50 copies/ml at switch to CAB+RPV LA or a new oral ART regimen between 21 January 2021 and 31 December 2022, and were active in care (i.e. ≥1 visit within 24 months before/at CAB+RPV LA or new oral ART regimen initiation). Only guideline‐recommended two‐ or three‐drug regimens were included in the oral ART group [20]. Individuals were followed from the date of their first CAB+RPV LA injection or new oral ART prescription until the first of (1) discontinuation of CAB+RPV LA or new oral ART regimen, (2) death, (3) loss to follow‐up (12 months after last clinical contact) or (4) study end (30 Ju2023; allowing for a potential of ≥6 months of follow‐up).

### 2.2 Measurements

Baseline demographic characteristics (age, sex, race, ethnicity, US geographic region) and clinical characteristics (injection drug use [IDU], history of syphilis, history of AIDS‐defining events [ADEs], VL, CD4 cell count and VACS scores) were obtained from EHRs, using the last entry at or before regimen initiation. Baseline comorbidities were assessed within 12 months before or at regimen initiation. Comorbidities were identified in the EHR using either diagnosis field searches or lab results, and consisted of autoimmune disease, cardiovascular disease, invasive cancer, endocrine disorder, mental health disorder, liver disease, bone disorder, peripheral neuropathy, renal disease, hypertension or substance use disorder. Prior core agent class and months on prior ART regimen (all participants) and specific oral ART regimen (oral ART group only) were obtained from prescriptions in the EHRs.

CAB+RPV LA discontinuation was defined as >67 days without injections (monthly dosing) or >127 days without injections (every other month dosing), based on the pharmacokinetic information from the label [2, 3]. Oral ART regimen discontinuation was defined as either ≥45 days without a prescription, or the start of a prescription for another regimen. A 45‐day grace period is often used in pharmacoepidemiologic studies to allow for late starts, missed doses stockpiled and the use of samples. In addition, a recent study examined different potential definitions (45, 60 and 90 days) and found that individuals who had different minimum interruption durations were both similar at baseline and experienced similar virologic outcomes over follow‐up [21]. CVF was assessed among those with at least one VL available during follow‐up and was defined as two consecutive VL ≥200 copies/ml or one VL ≥200 copies/ml followed by discontinuation within 4 months.

### 2.3 Statistical analyses

Demographic and clinical characteristics as well as outcomes were summarized using medians with interquartile ranges (IQRs) for continuous variables and frequencies with proportions for categorical variables. Pearson's chi‐square tests were used to compare categorical outcomes across regimens, and Mann−Whitney tests were used to compare continuous outcomes across regimens.

Among all individuals, time on regimen was summarized, along with the number who were on regimen at study end and the numbers who discontinued, died, and were lost to follow‐up during the study period. Among those who discontinued at any point, virologic suppression (VL < 200 copies/ml) at discontinuation was assessed, and the number who returned to the regimen of interest after discontinuation was described. The last VL available for each individual was summarized in terms of achieving VL < 200 copies/ml and VL < 50 copies/ml. Among individuals who experienced CVF, ART regimens following CVF were assessed, along with subsequent virologic suppression to < 200 and < 50 copies/ml at any time (among individuals with at least one VL available after CVF).

Unadjusted and adjusted logistic regression was used to assess the association between regimen type (CAB+RPV LA vs. oral ART) and CVF. Adjusted analyses controlled for age (linear and quadratic terms), sex, race, IDU, history of ADE, CD4 cell count (linear and quadratic terms), presence of comorbid conditions and prior regimen class.

In those receiving CAB+RPV LA, univariable logistic regression models were fit to evaluate potential predictors of CVF. Age, sex, race, US region, IDU, history of ADE, CD4 cell count (per 100 cells/µl), presence of comorbid conditions, prior regimen class and body mass index were evaluated.

## 3 RESULTS

### 3.1 Study population

A total of 1362 adults with VL < 50 copies/ml switched to CAB+RPV LA regimen, and 2783 switched to a new oral ART regimen during the study period (Table 1). Prior to CAB+RPV LA regimen or a new oral ART regimen, the most frequently prescribed regimens were bictegravir/emtricitabine/tenofovir alafenamide fumarate (BIC/FTC/TAF; 48%) and elvitegravir/cobicistat/emtricitabine/tenofovir alafenamide fumarate (34%), respectively (Table S1). Among individuals in the new oral ART group, 2593 (92%) were prescribed a second‐generation INSTI‐based two‐ or three‐drug single tablet regimen (STR); 58% received BIC/FTC/TAF, and 34% received a DTG‐based two‐ or three‐drug regimen (Table S2).

**Table 1:** Demographic and clinical characteristics at regimen start

	CAB+RPV LA *N* = 1362	Oral ART *N* = 2783
Age, median years (IQR)	39 (32, 52)	45 (34, 56)
Female sex, *n* (%)	237 (17)	514 (18)
Black race, *n* (%)^a^	557 (41)	1198 (43)
Hispanic ethnicity, *n* (%)^a^	390 (29)	678 (24)
Care in Southern USA, *n* (%)	752 (55)	1742 (63)
Injection drug use, *n* (%)	38 (3)	116 (4)
History of syphilis, *n* (%)	604 (44)	1134 (41)
History of AIDS‐defining event, *n* (%)	333 (24)	814 (29)
At least one comorbidity^b^	1086 (80)	2378 (85)
Viral load, median copies/ml (IQR)	19 (19, 20)	19 (19, 19)
CD4 cell count, median cells/µl (IQR)^a^	686 (496, 902)	700 (524, 913)
VACS index, median (IQR)	10 (0, 18)	11 (0, 22)
Prior core agent class, *n* (%)		
INSTI‐based	1003 (74)	1880 (68)
NNRTI‐based	106 (8)	474 (17)
PI‐based	42 (3)	203 (7)
More than one core agent	211 (16)	226 (8)
Months on prior ART regimen, median (IQR)	20 (7, 38)	37 (20, 55)

Abbreviations: AIDS, acquired immunodeficiency syndrome; ART, antiretroviral therapy; CAB+RPV LA, cabotegravir + rilpivirine long‐acting; INSTI, integrase inhibitor; IQR, interquartile range; *n*, number; NNRTI, non‐nucleoside reverse transcriptase inhibitor; PI, protease inhibitor; USA, United States of America; VACS, Veterans Aging Cohort Study.

^a^ *N* missing = 133 (race), 132 (ethnicity), 35 (CD4 cell count).

^b^ At least one of the following comorbidities (ever): autoimmune disease, cardiovascular disease, invasive cancer, endocrine disorder, mental health disorder, liver disease, bone disorder, peripheral neuropathy, renal disease, hypertension or substance use disorder.

Compared to oral ART users, CAB+RPV LA users were younger (aged ≥50 years: 29% vs. 41%), had been on their prior regimen for a shorter period (20 vs. 37 months), were more likely to switch from an INSTI (74% vs. 68%), were less likely to have comorbidities (80% vs. 85%); median CD4 cell counts at initiation were similar (686 [IQR: 496−902] vs. 700 [IQR: 524−913] cells/µl; Table 1). Overall, specific comorbidities were similarly distributed across groups; however, CAB+RPV LA users were somewhat less likely to have endocrine disorders (49% vs. 54%), liver disease (12% vs. 15%) or hypertension (31% vs. 35%), and they were somewhat more likely to have mental health disorders (42% vs. 39%; Table S3).

### 3.2 Persistence

CAB+RPV LA users were followed for a median of 11 months (IQR: 8, 14), whereas oral ART users were followed for a median of 17 months (IQR:12, 24; *p* < 0.001; Table 2). At study end, 80% of CAB+RPV LA users and 81% of oral ART users remained on their respective regimens (*p* = 0.59; Table 2). CAB+RPV LA users were more likely to discontinue their regimen than oral ART users (26% vs. 16%; *p* < 0.001); Table 2). CAB+RPV LA users who discontinued their regimen did so earlier than oral ART users (median [IQR] of 6 months [3, 10] vs. 8 months [3, 15]; *p* = 0.04) and were more likely to be virologically suppressed at discontinuation (80% vs. 65%; *p* < 0.001; Table 2). A similar proportion across groups (28% vs. 30%) returned to the regimen of interest (Table 2).

**Table 2:** Persistence

	CAB+RPV LA (*N* = 1362)	Oral ART (*N* = 2783)	*p*‐value
Months on regimen, median (IQR)	11 (8, 14)	17 (12, 24)	<0.001
Discontinued regimen, *n* (%)	350 (26)	453 (16)	<0.001
Months on regimen for discontinuers, median (IQR)	6 (3, 10)	8 (3, 15)	0.04
Virologically suppressed at discontinuation, *n* (%)	279 (80)	293 (65)	<0.001
Returned to regimen of interest, *n* (%)	98 (28)	136 (30)	0.53
On regimen of interest at study end, *n* (%)	1094 (80)	2255 (81)	0.59
Lost to follow up, *n* (%)	≤5^a^	127 (5)	<0.001
Died, *n* (%)	≤5^a^	6 (0)	0.74

Abbreviations: ART, antiretroviral therapy; CAB+RPV LA, cabotegravir + rilpivirine long‐acting; IQR, interquartile range; *n*, number.

^a^ HIPAA regulations require the masking of cells with 1−5 individuals.

### 3.3 Virologic outcomes

A median of 3 VLs was observed over follow‐up for both groups (IQR: 2, 4 for CAB+RPV LA; IQR: 2, 5 for oral ART; Table 3). High levels of virologic control were observed at last VL with injectable CAB+RPV LA (95% had VL < 50 copies/ml, 99% had VL < 200 copies/ml) and new oral ART (91% had VL < 50 copies/ml, 96% had VL < 200 copies/ml; Table 3). A total of 15 (1%) individuals on CAB+RPV LA regimen and 44 (2%) individuals on oral ART regimens experienced CVF. Among the 15 CVFs in CAB+RPV LA users, 11 (73%) had two consecutive follow‐up VL ≥200 copies/ml, and four (27%) had one follow‐up VL ≥200 copies/ml + discontinuation. Among the 44 CVFs in new oral ART users, 31 (70%) had two consecutive follow‐up VL ≥200 copies/ml and 13 (30%) had one follow‐up VL ≥200 copies/ml + discontinuation.

**Table 3:** Virologic outcomes among those with follow‐up viral load

		CAB+RPV LA *N* = 1293	Oral ART *N* = 2523
	Number of VLs available over follow‐up, median (IQR)	3.0 (2.0, 4.0)	3.0 (2.0, 5.0)
Last viral load	< 200 copies/ml, *n* (%)	1281 (99)	2431 (96)
	< 50 copies/ml, *n* (%)	1229 (95)	2298 (91)
CVF	*n* (%)	15 (1)	44 (2)
	Unadjusted odds ratio (95% CI)^a^	0.66 (0.35, 1.00)	Reference
	Adjusted odds ratio (95% CI)^a^, ^b^	0.64 (0.40, 1.02)	Reference

Abbreviations: ART, antiretroviral therapy; CAB+RPV LA, cabotegravir + rilpivirine long‐acting; CI, confidence interval; CVF, confirmed virologic failure; ml, millilitre; *n*, number.

^a^ Excluding 148 individuals missing race or baseline CD4 cell count.

^b^ Adjusted for age (linear and quadratic terms), female sex, Black race, injection drug use, history of AIDS‐defining events, CD4 cell count (linear and quadratic terms), presence of comorbidities and core class in prior regimen.

**Figure 1:**
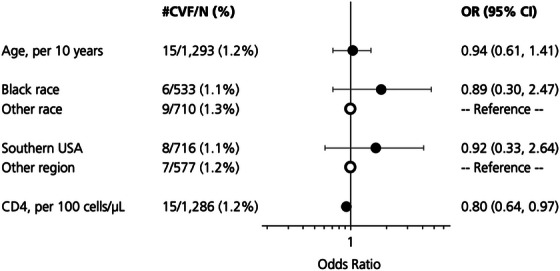
Predictors of confirmed virologic failure among people switching to CAB+RPV LA with ≥1 follow‐up viral load (*N* = 1293).^a^ Abbreviations: CAB+RPV LA, cabotegravir + rilpivirine long‐acting; CVF, confirmed virologic failure; *n*, number; OR, odds ratio; US, United States. ^a^Sex, body mass index, injection drug use, history of AIDS‐defining illnesses, comorbidities and core agent class of prior regimen were evaluated as potential predictors of CVF but not included in the multivariable model, as one subgroup per variable had ≤ 5 CVFs. ^b^ *N* missing = 50 for race, seven for CD4 cell count.

Among individuals with a follow‐up VL (CAB+RPV LA: *n* = 1236; oral ART: *n* = 2432), risk of CVF did not statistically differ (adjusted OR: 0.64 for CAB+RPV LA vs. oral ART; 95% CI: 0.34, 1.41; Table 3). Among CAB+RPV LA users, only baseline CD4 cell count marginally predicted CVF; every 100 CD4 cells/µl increase was associated with 20% lower risk of CVF, with an OR of 0.80 (95% CI: 0.64, 1.97; Figure 1).

Of the 15 individuals with CVF on CAB+RPV LA, more than half (53%) went on to an INSTI‐based oral regimen. Of note, more than 25% went on to regimens containing at least two core agents. Of the nine with VL after CVF, 89% achieved a VL < 200 copies/ml and 67% achieved a VL < 50 copies/ml after CVF.

Of the 44 individuals with CVF on oral therapy, the majority stayed on the same regimen (52%) or went on to an INSTI‐based oral regimen (34%); the remainder either remained off therapy or went on a variety of other regimens. Of the 15 with VL after CVF, 67% achieved < 200 copies/ml and 53% achieved < 50 copies/ml after CVF.

## 4 DISCUSSION

This study aimed to compare outcomes in people with HIV with VL < 50 copies/ml who received CAB+RPV LA injections to those prescribed a new guidelines‐recommended oral ART regimen over the first 2 years of CAB+RPV LA availability, as well as to assess potential predictors of CVF among CAB+RPV LA users. Observations from the first 2 years of CAB+RPV LA availability (median of 11 and 17 months of follow‐up for CAB+RPV LA and oral ART users, respectively) suggest that CAB+RPV LA injectable is as effective as a switch to modern oral ART (92% of patients were on second‐generation STR INSTIs). While CAB+RPV LA users were more likely to discontinue and to discontinue earlier, they were more likely to be virologically suppressed at discontinuation. High levels of virologic suppression (VL < 50 copies/ml) were observed at last VL with both CAB+RPV LA (95%) and new oral ART (91%); and risk of CVF did not differ between individuals switching to CAB+RPV LA or new oral ART regimens (1% vs. 2%; adjusted OR [95% CI] = 0.64 [0.34, 1.14]). Lower CD4 cell count at initiation was the only predictor of CVF with CAB+RPV LA. Of CAB+RPV LA users with CVF, more than half (53%) switched to an INSTI oral therapy, while those on oral therapy were most likely to maintain their current regimen (52%) after CVF.

Clinical trials have demonstrated the safety and efficacy of CAB+RPV LA injectable (both monthly and every 2‐months dosing) when compared to daily oral three‐drug regimens [5−12]. Recent observational studies have found that most individuals on the CAB+RPV LA regimen achieve or maintain virologic suppression and that CVF is rare [17−19]. A previous analysis of the OPERA cohort in the United States found that among 237 virologically suppressed individuals starting CAB+RPV LA, most injections were administered on time and that high levels of virologic control were achieved, with 95% having VLs < 50 copies/ml and 99% having VLs < 200 copies/ml at last VL [19]. In addition, a nationwide observational study within the Swiss HIV Cohort Study found that of 186 individuals with HIV on CAB+RPV LA from March 2022 to March 2023, 172 (92%) maintained VLs < 50 copies/ml and three (2%) experienced CVF [17]. Furthermore, a single‐centre study at the IMSUT Hospital in Tokyo found that of 74 individuals who received CAB+RPV LA, more than 97% maintained VL < 50 copies/ml at all visits up to 13 months after their first CAB+RPV LA injection; no individuals experienced CVF [18].

In this real‐world analysis, we similarly found high levels of virologic control after switching to CAB+RPV LA. However, no published observational studies have directly compared the effectiveness of a switch to CAB+RPV LA to that of a new oral regimen. This study showed that the risk of CVF was not statistically different between switches to CAB+RPV LA or a new oral ART. Of note, 92% of regimens in the oral ART group comparator included a second‐generation INSTI STR, which are the standard of treatment; as single tablets, they are the most clinically relevant comparison to a complete LA ART regimen such as CAB+RPV LA. Although it has been shown that elderly individuals may be at lower risk for suboptimal drug concentrations, age was not associated with CVF among CAB+RPV LA users in this study [22]. Lower CD4 cell counts, however, were associated with CVF; this is not surprising given that CD4 cell count and VL are inversely correlated.

This study was one of the first studies to examine CAB+RPV LA outcomes in a real‐world setting in the United States and included a large population of new CAB+RPV LA users. It was also the first study in the United States to compare CAB+RPV LA to a switch to modern oral ART in routine clinical care. Nonetheless, there are several limitations to consider. This study was limited by the lack of data on adherence, resistance and reasons for discontinuation. The slow uptake of the CAB+RPV LA regimen in the early months of approval resulted in less time available for follow‐up for the CAB+RPV LA group and thus a longer follow‐up period for the oral ART group. Adherence was not an outcome for this study, as it could not be assessed in the oral ART group; however, a previous study on CAB+RPV LA adherence in OPERA found that 23% of CAB+RPV LA users experienced at least one delay, with a median of 1 delay among individuals with delays [19]. Documentation of exact dates for discontinuation for both injectables and oral ART was variable and often missing. We used the pharmacokinetic information from the label to determine when a patient no longer had therapeutic levels of CAB+RPV if no additional injections were given to determine discontinuation. Similarly, as is the case with most observational studies, prescriptions and events that occurred outside of OPERA may not have been documented and, therefore, not accounted for in the analyses. While CAB+RPV LA users were more likely to discontinue than oral ART users, they were similarly likely to be on their respective regimen at study end. This may possibly have been due to oral bridging; however, bridging is regrettably infrequently documented in EHR, thus this hypothesis could not be tested. Incidence rates could not confidently be calculated for outcomes of interest (discontinuation, CVF) because the nature of prescription and injection data differ. While injections are directly observed therapy with documented dates of administration, prescriptions may have lags in filling, ingestion and absorption, or may not be taken at all. In addition, VL testing frequency differed between the groups (same median number of follow‐up VLs over a shorter follow‐up period for the CAB+RPV LA group vs. the oral ART group), as individuals visiting clinics for their injections are more likely to be tested and to be tested sooner [23]. Differential monitoring across groups was addressed by using logistic regression as opposed to Cox proportional hazards. Finally, although we adjusted for multiple variables when assessing the association between regimen type (CAB+RPV LA vs. oral ART) and CVF, it is possible that some residual confounding across groups remained.

## 5 CONCLUSIONS

This real‐world assessment demonstrated high levels of virologic control among virologically suppressed people with HIV who switched to CAB+RPV LA or a new predominantly second‐generation single tablet INSTI‐based oral ART regimen in the United States. The risk of CVF did not differ between individuals switching to CAB+RPV LA or oral ART regimens. Lower CD4 count at initiation was the only predictor of CVF with CAB+RPV LA. At study end, a similar proportion remained on CAB+RPV LA and new oral ART regimens. Therefore, CAB+RPV LA may be an appropriate option for virologically suppressed individuals and appears to be as effective as daily oral therapy to control HIV.

## COMPETING INTERESTS

RKH has received research grants from Gilead Sciences, Janssen and ViiV Healthcare, speaker honoraria from ViiV Healthcare, Merck, Gilead Sciences and Janssen, and advisory board participation with ViiV Healthcare, Gilead Sciences, Janssen and Epividian. MGS is on the Speakers Bureau for ViiV Healthcare and Gilead Sciences, and on the advisory board for ViiV Healthcare and Epividian. JSF, LB, QC, BL and GPF are employed by Epividian, Inc.; Epividian has had research funded by the AIDS Healthcare Foundation, EMD Serono, Gilead Sciences, Janssen Scientific Affairs, LLC, Merck & Co., Theratechnologies Inc. and ViiV Healthcare. GS, VV and JVW are employed by ViiV Healthcare and hold stocks and shares in GSK as part of their employment. MBW has participated in post‐conference advisory boards for the Conference on Retroviruses and Opportunistic Infections (CROI) and International AIDS Conference (IAC) and also serves as a principal investigator on ViiV Healthcare clinical trials, but does not receive personal compensation for this work, which goes directly to the AIDS Healthcare Foundation. MBW is also a member of the Epidemiology and Clinical Advisory Board for Epividian.

## AUTHOR CONTRIBUTIONS

JSF and LB share the responsibility for the design of this study. RKH, MGS, JSF, QC, MBW and GPF contributed to the acquisition of data. JSF, LB and BL performed all analyses. All authors contributed to the interpretation of results. JSF and BL drafted the manuscript. All authors have critically reviewed and approved the manuscript and have participated sufficiently in the work to take public responsibility for its content.

## FUNDING

This work was funded by ViiV Healthcare.

## ETHICAL APPROVAL STATEMENT

OPERA complies with all HIPAA and HITECH requirements, which expand upon the ethical principles detailed in the 1964 Declaration of Helsinki. OPERA has received annual institutional review board (IRB) approval by Advarra IRB, including a waiver of informed consent and authorization for use of protected health information (Pro00023648).

## PRIOR PRESENTATION

Parts of the data were presented at the following conference: CROI 2024 (Denver, CO, USA, March 3–6, 2024).

## Supporting information




**Table S1**. Top 10 most frequently prescribed regimens prior to CAB+RPV LA injections or oral ART regimens
**Table S2**. Frequency of ART regimens in the oral ART group
**Table S3**. Baseline comorbid conditions

## Data Availability

The datasets used in this study are not publicly available due to privacy concerns and the proprietary nature of the database, but can be accessed upon reasonable request through the corresponding author to the OPERA Epidemiological and Clinical Advisory Board.
