# Supplementary material for: Comparable real‐world effectiveness between switches to cabotegravir + rilpivirine long‐acting or modern daily oral regimens in the United States: an OPERA cohort study
Source: J Int AIDS Soc. 2025 Dec 17;28(12):e70068. doi: 10.1002/jia2.70068 (PMC12710068; doi:10.1002/jia2.70068)
Supplement: Supplementary file 1 — Table S1. Top 10 most frequently prescribed regimens prior to CAB+RPV LA injections or oral ART regimens Table S2. Frequency of ART regimens in the oral ART group Table S3. Baseline comorbid conditions [file JIA2-28-e70068-s001.docx]

Supplementary Material

**Supplementary Table 1.** Top 10 most frequently prescribed regimens prior to CAB+RPV LA injections or oral ART regimens

**Supplementary Table 2.** Frequency of ART regimens in the oral ART group

**Supplementary Table 3**. Baseline comorbid conditions

**Supplementary Table 1.** Top 10 most frequently prescribed regimens prior to CAB+RPV LA injections or oral ART regimens

|  | **Number (%)** | **Cumulative Percent** |
| --- | --- | --- |
|  | | |
| **Regimens prior to switch to CAB+RPV LA injections** | **1362 (100)** | **100** |
| Bictegravir/emtricitabine/tenofovir alafenamide fumarate | 652 (48) | 48 |
| Dolutegravir/lamivudine | 148 (11) | 59 |
| Dolutegravir/abacavir/lamivudine | 130 (10) | 68 |
| Elvitegravir/cobicistat/emtricitabine/tenofovir alafenamide fumarate | 111 (8) | 76 |
| Rilpivirine/emtricitabine/tenofovir alafenamide fumarate | 82 (6) | 82 |
| Darunavir/cobicistat/emtricitabine/tenofovir alafenamide fumarate | 49 (4) | 86 |
| Dolutegravir/rilpivirine | 43 (3) | 89 |
| Dolutegravir/emtricitabine/tenofovir alafenamide fumarate | 19 (1) | 91 |
| Dolutegravir/rilpivirine/emtricitabine /tenofovir alafenamide fumarate | 11 (1) | 91 |
| Darunavir/cobicistat/dolutegravir | 9 (1) | 92 |
| Any other regimen | 108 (8) | 100 |
|  | | |
| **Regimens prior to switch to oral regimens (N=2,783)** | **2783 (100)** | **100** |
| Elvitegravir/cobicistat/emtricitabine/tenofovir alafenamide fumarate | 933 (34) | 34 |
| Bictegravir/emtricitabine/tenofovir alafenamide fumarate | 433 (16) | 49 |
| Rilpivirine/emtricitabine/tenofovir alafenamide fumarate | 216 (8) | 57 |
| Dolutegravir/abacavir/lamivudine | 215 (8) | 65 |
| Dolutegravir/emtricitabine/tenofovir alafenamide fumarate | 203 (7) | 72 |
| Efavirenz/emtricitabine/tenofovir disoproxil fumarate | 155 (6) | 77 |
| Darunavir/cobicistat/emtricitabine/tenofovir alafenamide fumarate | 135 (5) | 82 |
| Elvitegravir/cobicistat/emtricitabine/tenofovir disoproxil fumarate | 48 (2) | 84 |
| Dolutegravir/lamivudine | 44 (2) | 86 |
| Raltegravir/emtricitabine/tenofovir alafenamide fumarate | 35 (1) | 87 |
| Any other regimen | 366 (13) | 100 |

**Supplementary Table 2.** Frequency of ART regimens in the oral ART group

| **Regimen Type** | **Oral ART (N = 2,783)**  **n (%)** |
| --- | --- |
| bictegravir/emtricitabine/tenofovir alafenamide | 1636 (58) |
| dolutegravir/lamivudine | 929 (33) |
| darunavir/cobicistat/emtricitabine/tenofovir alafenamide | 109 (4) |
| doravirine/lamivudine/tenofovir disoproxil fumarate | 52 (2) |
| rilpivirine/emtricitabine/tenofovir alafenamide | 41 (1) |
| elvitegravir/cobicistat/emtricitabine/tenofovir alafenamide | 30 (1) |
| dolutegravir/lamivudine/abacavir | 28 (1) |
| Other regimens | ≤ 5^a^ |

^a^ HIPAA regulations require the masking of cells with 1 to 5 individuals

**Supplementary Table 3.** Baseline comorbid conditions

| **Comorbidity** | **CAB+RPV LA (N=1,362)**  **n (%)** | **Oral ART (N=2,783)**  **n (%)** |
| --- | --- | --- |
| Autoimmune Disease^a^ | 53 (4) | 83 (3) |
| Cardiovascular Disease^b^ | 98 (7) | 177 (6) |
| Invasive Cancers | 62 (5) | 127 (5) |
| Endocrine Disorders^c^ | 661 (49) | 1,504 (54) |
| Mental Health Disorders^d^ | 566 (42) | 1,081 (39) |
| Liver Disease^e^ | 167 (12) | 416 (15) |
| Bone Disorders^f^ | 66 (5) | 137 (5) |
| Peripheral Neuropathy | 157 (12) | 317 (11) |
| Renal Disease^g^ | 172 (13) | 324 (12) |
| Hypertension | 419 (31) | 986 (35) |
| Substance Abuse^h^ | 296 (22) | 632 (23) |

^a^ Autoimmune disease: rheumatoid arthritis, psoriasis, ankylosing spondylitis, multiple sclerosis, myasthenia gravis, lupus, Hashimoto’s thyroiditis, Grave’s disease, ulcerative colitis, Crohn’s disease, inflammatory bowel disease, chronic inflammatory demyelinating polyneuropathy (CIDP), Guillain-Barre syndrome, Sjögren’s syndrome, vasculititis
^b^ Cardiovascular disease: myocardial infarction, angina, transient ischemic attack, stroke, other/unspecified CHD, occlusion/stenosis of precerebral arteries, other CBV, peripheral arterial disease, abdominal aortic aneurysm
^c^ Endocrine disorders: diabetes mellitus, hyperlipidemia, hypothyroidism, hyperthyroidism
^d^ Mental health disorder: anxiety disorder, dissociative and conversion disorders, organic psychotic conditions (dementia), other psychoses, mood disorders, somatoform disorders, pervasive developmental disorders, suicidality
^e^ Liver disease: NAFLD, NASH, cirrhosis, steaotohepatitis, hepatitis E, hepatitis, hepatopathy
^f^ Bone disorder: osteopenia, osteoporosis
^g^ Renal disease: diagnosis of kidney disease, kidney failure, kidney injury, chronic kidney disease, renal insufficiency, end stage renal disease
^h^ Substance abuse: alcohol or drug abuse/dependence/disorder
